# Effect of Recombinant Vesicular Stomatitis Virus–Zaire Ebola Virus Vaccination on Ebola Virus Disease Illness and Death, Democratic Republic of the Congo

**DOI:** 10.3201/eid2806.212223

**Published:** 2022-06

**Authors:** Neil Rupani, Mbong Eta Ngole, J. Austin Lee, Adam R. Aluisio, Monique Gainey, Shiromi M. Perera, Lina Kashibura Ntamwinja, Ruffin Mbusa Matafali, Rigo Fraterne Muhayangabo, Fiston Nganga Makoyi, Razia Laghari, Adam C. Levine, Alexis S. Kearney

**Affiliations:** Brown University, Providence, Rhode Island, USA (N. Rupani, J.A. Lee, A.R. Aluisio, A.C. Levine, A.S. Kearney);; International Medical Corps, Goma, Democratic Republic of the Congo (M.E. Ngole, L.K. Ntamwinja, R.M. Matafali, R.F. Muhayangabo, F.N. Makoyi, R. Laghari);; Rhode Island Hospital, Providence (M. Gainey);; International Medical Corps, Washington, DC, USA (S.M. Perera)

**Keywords:** Ebola virus, recombinant vesicular stomatitis virus–Zaire Ebola virus, rVSV-ZEBOV, viruses, hemorrhagic fever, Ebola, EVD, Ebola vaccines, disease outbreaks, vector-borne infections, illness, death, vaccines, zoonoses, Democratic Republic of the Congo

## Abstract

We conducted a retrospective cohort study to assess the effect vaccination with the live-attenuated recombinant vesicular stomatitis virus–Zaire Ebola virus vaccine had on deaths among patients who had laboratory-confirmed Ebola virus disease (EVD). We included EVD-positive patients coming to an Ebola Treatment Center in eastern Democratic Republic of the Congo during 2018–2020. Overall, 25% of patients vaccinated before symptom onset died compared with 63% of unvaccinated patients. Vaccinated patients reported fewer EVD-associated symptoms, had reduced time to clearance of viral load, and had reduced length of stay at the Ebola Treatment Center. After controlling for confounders, vaccination was strongly associated with decreased deaths. Reduction in deaths was not affected by timing of vaccination before or after EVD exposure. These findings support use of preexposure and postexposure recombinant vesicular stomatitis virus–Zaire Ebola virus vaccine as an intervention associated with improved death rates, illness, and recovery time among patients with EVD.

Ebola virus disease (EVD) has ranked among the deadliest of all infectious diseases since its documented emergence during 1976 in Zaire (now the Democratic Republic of the Congo; DRC) (*1*). Since 1976, there have been 41 EVD outbreaks, most of which have occurred in sub-Saharan Africa. Case-fatality rates have ranged from 25% to 90% in these outbreaks (*2*). EVD is caused by 1 of 5 species of *Ebolavirus* that are known to infect humans. Symptom onset occurs ≈10 days after exposure and commonly includes malaise, myalgias, fever, nausea, vomiting, diarrhea, rash, and bleeding (*1*). EVD is transmitted through body fluids, which enables the disease to spread through direct, close contact (*3*). Historically, supportive care, such as fluid and electrolyte repletion, has been the most effective treatment for EVD (*1*). However, EVD thrives in areas where poverty and inadequate healthcare infrastructure intersect, limiting the ability to rapidly diagnose cases or provide adequate supportive care (*4*).

The deadliest EVD outbreak was the 2014–2016 West Africa outbreak, which had 28,610 cases and 11,308 deaths (*2*). The sheer size and subsequent socioeconomic effect of this outbreak sparked an unprecedented effort to develop and study new treatment and prevention strategies for EVD, including randomized clinical trials of Ebola virus vaccinations (*5*). The recombinant vesicular stomatitis virus–Zaire Ebola virus (rVSV-ZEBOV) vaccine, known commercially as Ervebo, is a live-attenuated recombinant vesicular stomatitis virus vaccine. It is administered as a single-dose intramuscular injection (*6*). It is effective against the species *Zaire ebolavirus* (ZEBOV), but does not protect against other species of *Ebolavirus* (*7*). The rVSV-ZEBOV vaccine was initially administered in Guinea under emergency use authorization by the US Food and Drug Administration (FDA) and in DRC under compassionate use by the World Health Organization (WHO) (*8**,**9*).

Multiple clinical trials have demonstrated that the rVSV-ZEBOV vaccine is well tolerated without serious adverse events. However, many vaccine recipients report self-limiting systemic symptoms, including fever, headache, myalgias, and fatigue, within the first 24 hours after vaccination. Symptoms caused by reactogenicity mimic the first symptoms of EVD; this reaction is essential to consider, particularly in outbreak settings, because recipients are often vaccinated after a potential EVD exposure. Vaccine recipients have also reported delayed side effects, including polyarthralgia, polyarticular arthritis, and skin eruptions in the first 2–3 weeks after vaccination (*10**–**12*). Clinical trials have demonstrated that the vaccine is highly immunogenic, elicits immune responses that are largely maintained over a 12-month period, and is highly effective at preventing EVD (*10**,**13**–**15*).

On August 1, 2018, the DRC Ministry of Health declared its 10th EVD outbreak, which became the second deadliest in history, resulting in 3,481 cases and 2,299 deaths (*2**,**9*). The *Zaire ebolavirus* species was identified as the cause of the outbreak (*16*). A ring vaccination strategy was implemented to administer rVSV-ZEBOV vaccine during this outbreak, targeting contacts of cases, contacts of contacts, and healthcare workers (*9*). Many persons were vaccinated postexposure. Other persons might have received preexposure vaccination, particularly if they were identified as contacts of contacts. In late 2019, rVSV-ZEBOV vaccine was prequalified by WHO and approved for use in persons >18 years of age by the FDA (*17**,**18*). To date, >350,000 persons have received rVSV-ZEBOV vaccine in Guinea and the DRC (*2*). Although studies have demonstrated the vaccine is safe and effective, WHO states that further research is needed to support its full licensure (*19*).

A major remaining question is whether rVSV-ZEBOV vaccine can reduce illness and death for patients who have confirmed EVD, in addition to preventing infection. Other vaccines, such as those directed against pertussis, varicella, and rotavirus, have evidence supporting reduced illness, death, and disease severity in patients experiencing breakthrough infections (*20*,*21*). More recently, vaccination against SARS-CoV-2 with authorized mRNA vaccines has demonstrated reduced viral load, lower risk for febrile symptoms, and shorter duration of symptoms among persons experiencing breakthrough infections (*22*). Furthermore, some vaccines have been shown to provide protection when administered after exposure. Examples include measles, rabies, hepatitis A and B, and varicella vaccines (*20*). The purpose of our study was to determine the effect that vaccination with rVSV-ZEBOV has on clinical characteristics and outcomes among patients with laboratory-confirmed EVD.

## Methods

### Study Design, Setting, and Population

**Figure 1:**
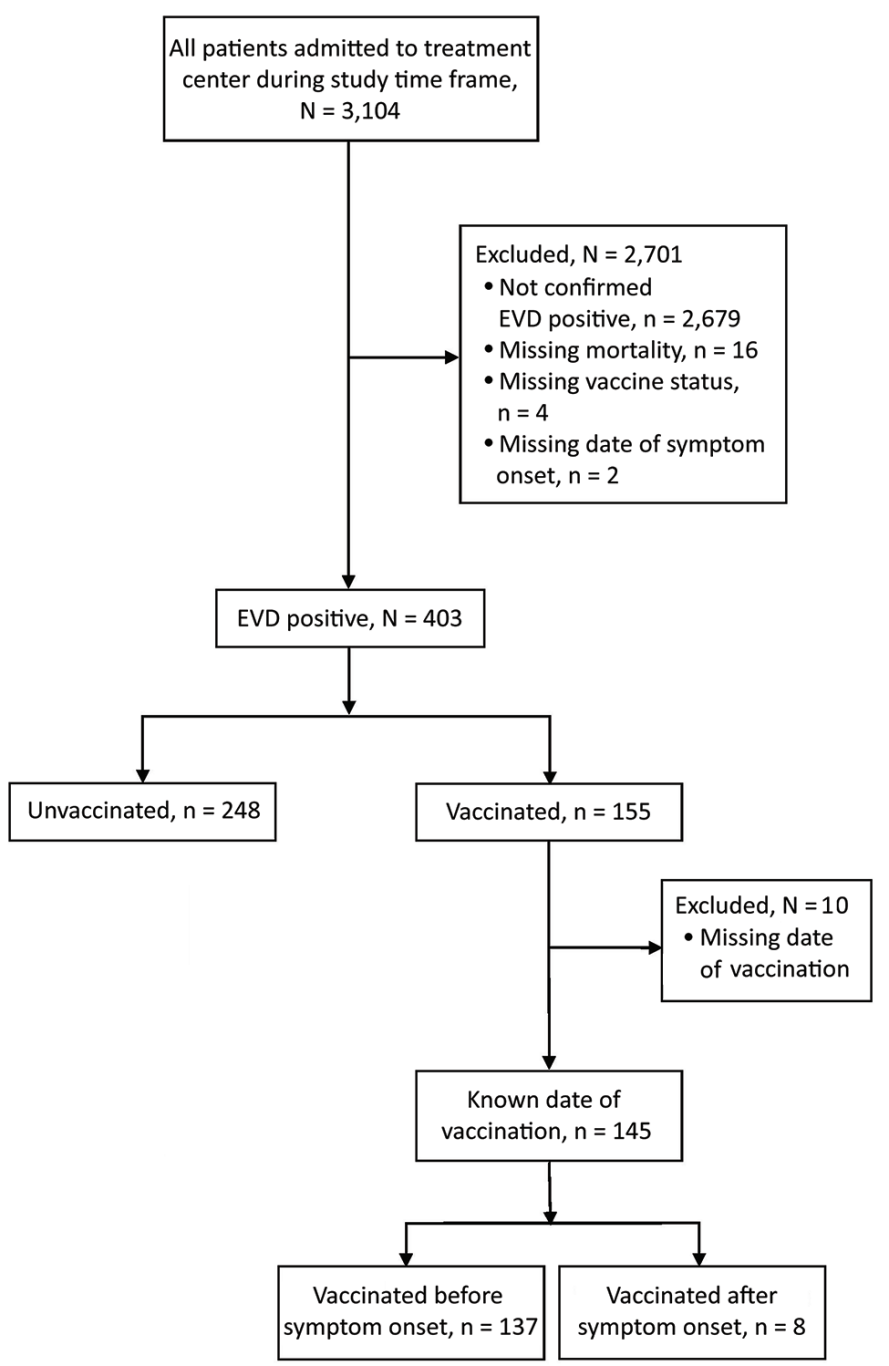
Inclusion/exclusion algorithm and makeup of study sample for study of impact of recombinant vesicular stomatitis virus–Zaire Ebola virus vaccination on EVD illness and death, Democratic Republic of the Congo. EVD, Ebola virus disease.

We conducted a retrospective cohort study of patients who came for care at the International Medical Corps Mangina Ebola Treatment Center (ETC) during the 2018 EVD outbreak in the DRC. The eastern provinces of the DRC (North Kivu and Ituri) served as the main catchment area for the Mangina ETC, located in North Kivu. All persons who came to the Mangina ETC during December 7, 2018‒January 29, 2020, who had laboratory-confirmed diagnosis of EVD were eligible for inclusion in this study. Persons were excluded if they did not have a documented EVD outcome (death or survival), if the patient’s vaccination status was unknown, or if they did not have a reported date of symptom onset (Figure 1). The Institutional Review Board at Rhode Island Hospital (Lifespan Health System, Providence, RI, USA) provided ethics exemption for this study and waived the requirement to obtain informed consent.

### Laboratory Diagnosis

All patients had laboratory testing conducted by the Institut National de Recherche Biomédicale (Kinshasa-Bombe, DRC). The Cepheid GeneXpert Ebola Assay (https://www.cepheid.com) was used for detection of ZEBOV RNAs encoding surface glycoprotein and nucleoprotein. The assay was also used to determine the cycle threshold (Ct), a proxy for viral load (*23*,*24*). The Ct value is inversely proportional to viral load; a Ct value >40 was considered negative for cases. A reverse transcription PCR was used to confirm EVD cases.

### Study Procedures

Response teams were deployed to health zones in North Kivu, South Kivu, and Ituri Provinces in the eastern part of DRC to identify suspected, confirmed, or probable cases of EVD. Suspected and confirmed case-patients were isolated and transported to ETCs for further testing and treatment. Patients could also self-present to the ETC. All patients were screened by trained clinical staff to ensure they met the clinical case definition for suspected or confirmed EVD based on WHO and Médecins Sans Frontières guidelines, in consultation with local health authorities (*25**,**26*). Patients who had a previously confirmed laboratory diagnosis of EVD were directly admitted to the confirmed ward. Patients who met the case definition for suspected EVD were admitted to the ETC suspect ward, in which blood samples were drawn for initial EVD testing. If the initial test result of the patient was negative, they remained in the ETC until 72 hours had passed since symptom onset, at which point a second test was performed. Patients with a positive test result at that point were considered EVD positive and moved to the confirmed ward for further management (*27*,*28*). All patients who died during admission to the suspect ward or were dead on arrival to the ETC had an oral swab specimen taken for PCR testing before being moved to the morgue.

During triage at the ETC, detailed information was collected about each patient on standardized clinical forms, which included demographics, symptoms, potential contact with a suspected or confirmed EVD individual, comorbidities, and self-reported Ebola vaccination status. During ETC admission, protocol based care was provided. Patients were discharged from the ETC after 2 consecutive negative laboratory test results. The Mangina ETC also served as a PALM Trial site (Pamoja Tulinde Maisha [Together Save Lives in Kiswahili]), in which patients were randomized to receive experimental therapeutics (*29*). Additional detailed information about the clinical care provided at the ETC is provided (Appendix).

### Data Management

Data were retrospectively abstracted from clinical documentation by independent trained study personnel blinded to the specific study aims and entered into a standardized digital database. Additional information on data management is provided (Appendix).

### Statistical Methods and Variables

We performed data analyses by using R Studio version 4.0.2 (*30*). We used a Pearson χ^2^ test and a Fisher exact test to measure association between categorical variables and the Wilcoxon rank-sum test for continuous variables. Significance was established at p value <0.05. We used a case-centered, multivariable logistic regression and the Cox Proportional-Hazards model to examine the association between previous vaccination with rVSV-ZEBOV (exposure of interest) and the primary outcome of facility-based death (*31*). Models controlled for potential confounders including age, sex, time between symptom onset and admission to the ETC, treatment with experimental therapeutic agents, and Ct value (inversely proportional to viral load). We incorporated age^2^ into models to control for the quadratic relationship between age and survival for EVD patients (*32*).

One variable included in our models accounted for the experimental therapeutics patients received. Previous research has demonstrated that, of the 4 potential therapeutics administered at the ETC, 2 of these treatments (monoclonal antibody [mAb] 114, a single mAb; and REGN-EB3, a triple mAb) are more effective against EVD than the other 2 treatments (Zmapp, a triple mAb; and Remdesivir, an antiviral agent) (*29*). As a result, this variable was categorized on the basis of whether the patient received mAb114 or REGN-EB3, Zmapp or Remdesivir, or no therapeutics.

Additional posthoc analysis explored the effect vaccination timing had on deaths. We used the Cox Proportional-Hazards model to analyze this relationship. In previous vaccine efficacy studies, EVD cases with a symptom onset >10 days from randomization were included in the analyses. This categorical cutoff was chosen to account for the incubation period for EVD (*33**,**34*), time between symptom onset and laboratory confirmation, and the unknown period of time between vaccination and vaccine-induced protective immunity (*15*). The typical incubation period for EVD is 10 days after exposure to the disease, although data suggest that it might be shorter for children (*33**,**34*). Therefore, in our subanalysis, we used vaccination >10 days before symptom onset as the categorical cutoff. We also used vaccination at 7 and 14 days before symptom onset as cutoffs in a sensitivity analysis. In addition, although we excluded persons who were vaccinated after symptom onset from our initial analysis, we conducted a separate sensitivity analysis examining the effect vaccination had on deaths within this smaller group.

## Results

### Characteristics of Study Participants

Of the 3,104 persons admitted to the Mangina ETC during December 7, 2018‒January 29, 2020, a total of 403 patients had laboratory-confirmed EVD. Of those, 385 patients had sufficient data for analysis; 137 (35.6%) had been vaccinated before onset of symptoms. An additional 8 patients were vaccinated after symptom onset; these patients were excluded from the initial analysis (Figure 1).

We outlined the similarities and differences between the unvaccinated and vaccinated groups (Table 1). Among EVD-confirmed case-patients, a larger proportion of unvaccinated persons were female (63.3%) than male (36.7%) (p = 0.018). Vaccinated patients came to the ETC earlier in their disease course than unvaccinated patients (2 vs. 5 days after symptom onset; p<0.001), were older (median age 28.0 years vs. 25.5 years; p = 0.044), and were more likely to have reported contact with a suspected or confirmed EVD-positive person (65.7% vs. 52.4%; p<0.001).

**Table 1:** Patient characteristics for study of the effect of recombinant vesicular stomatitis virus–Zaire Ebola virus vaccination on Ebola virus disease illness and death, Democratic Republic of the Congo*

Characteristic	Overall, n = 385	Not vaccinated, n = 248	Vaccinated, n = 137	p value†
Age, y	26.0 (18.0‒40.0)	25.5 (12.0‒40.0)	28.0 (20.0‒40.0)	**0.044**
<5	49 (12.7)	43 (17.3)	6 (4.4)	**0.001**
5‒15	34 (8.8)	26 (10.5)	8 (5.8)	
16‒25	101 (26.2)	55 (22.2)	46 (33.6)	
26‒35	88 (22.9)	54 (21.8)	34 (24.8)	
36‒45	38 (9.9)	19 (7.6)	19 (13.9)	
46‒55	41 (10.7)	27 (10.9)	14 (10.2)	
>55	34 (8.8)	24 (9.7)	10 (7.3)	
Sex				**0.018**
M	159 (41.3)	91 (36.7)	68 (49.6)	
F	226 (58.7)	157 (63.3)	69 (50.4)	
Province				**0.002**
North Kivu	235 (61.0)	142 (57.3)	93 (67.9)	
Ituri	142 (36.9)	104 (41.9)	38 (27.7)	
Unknown	8 (2.1)	2 (0.8)	6 (4.4)	
Known or suspected Ebola contact				**<0.001**
No	73 (19.0)	62 (25.0)	11 (8.0)	
Yes	220 (57.1)	130 (52.4)	90 (65.7)	
Unknown	92 (23.9)	56 (22.6)	36 (26.3)	
Days between symptom onset and admission, d	4.0 (2.0‒6.0)	5.0 (3.0‒7.0)	2.0 (1.0‒4.0)	**<0.001**
First cycle threshold value	21.6 (18.2‒26.2)	20.4 (17.7‒24.2)	24.6 (19.9‒28.1)	**<0.001**
Therapeutic received				**0.005**
None	65 (16.9)	53 (21.4)	12 (8.8)	
Zmapp or Remdesivir	76 (19.7)	46 (18.5)	30 (21.9)	
mAb114 or REGN-EB3	244 (63.4)	149 (60.1)	95 (69.3)	
Final outcome				**<0.001**
Died	191 (49.6)	157 (63.3)	34 (24.8)	
Survived	194 (50.4)	91 (36.7)	103 (75.2)	
Length of stay among survivors, d	21.0 (18.0‒26.0), n = 193	22.0 (19.0‒28.5), n = 91	20.0 (17.0‒23.8), n = 102	**0.004**

*Values are median (IQR) or no. (%) . IQR, interquartile range; MAb, monoclonal antibody. †Statistical tests were performed by using the Wilcoxon rank sum test, the Pearson χ^2^ test, and the Fisher exact test. Boldface indicates a significant difference (p<0.05).

Although the rVSV-ZEBOV vaccine is FDA approved for use in persons >18 years of age, some children received the vaccine through investigative protocols. A larger proportion of vaccinated patients were from North Kivu Province (67.9%) than from Ituri Province (27.7%) (p = 0.002). A total of 16 (10.2%) unvaccinated women were pregnant, and 10 (14.5%) vaccinated women were pregnant. Vaccinated persons were more likely to receive mAb114 or REGN-EB3 than were unvaccinated persons (69.3% vs. 60.1%). We provide additional information about specific anti-EBOV treatments stratified by vaccination timing. (Appendix Table 1).

### EVD-Associated Clinical Findings

A greater proportion of unvaccinated patients experienced EVD-associated symptoms than did vaccinated patients. These symptoms included nausea, diarrhea, asthenia, anorexia, abdominal pain, chest pain, myalgia, dyspnea, dysphagia, sore throat, conjunctivitis, and bleeding (Table 2).

**Table 2:** Frequency of symptoms reported by vaccinated and unvaccinated Ebola virus disease‒confirmed patients, Democratic Republic of the Congo

Symptom	Not vaccinated, n = 248, no. (%)	Vaccinated, n = 137, no. (%)	p value*
Asthenia†	214 (86.6)	102 (74.5)	0.004
Anorexia	204 (82.3)	80 (58.4)	**<0.001**
Fever	193 (77.8)	99 (72.3)	0.273
Headache†	156 (63.2)	90 (65.7)	0.700
Abdominal pain	152 (61.3)	56 (40.9)	**<0.001**
Nausea	140 (56.5)	50 (36.5)	**<0.001**
Conjunctivitis†	138 (55.9)	49 (35.8)	**<0.001**
Diarrhea†	137 (55.5)	45 (32.8)	**<0.001**
Arthralgia	134 (54.0)	74 (54.0)	1.000
Myalgia	128 (51.6)	54 (39.4)	**0.029**
Chest pain†	89 (36.0)	32 (23.4)	**0.014**
Cough†	75 (30.4)	36 (26.3)	0.466
Bleeding‡	67 (27.1)	14 (10.3)	**<0.001**
Dysphagia	61 (24.6)	17 (12.4)	**0.007**
Sore throat†	52 (21.1)	17 (12.4)	**0.048**
Dyspnea†	47 (19.0)	13 (9.5)	**0.020**
Coma‡	16 (6.5)	2 (1.5)	0.050
Confusion‡	14 (5.7)	2 (1.5)	0.090
Rash‡	14 (5.7)	5 (3.7)	0.540
Hiccup‡	14 (5.7)	3 (2.2)	0.188
Jaundice†	12 (4.9)	2 (1.5)	0.156
Photophobia†	5 (2.0)	1 (0.7)	0.588

*Boldface indicates a significant difference (p<0.05). †One patient had missing data for this symptom. ‡Two patients had missing data for this symptom.

### Diagnostic Testing and Time to First Negative Test Result

**Figure 2:**
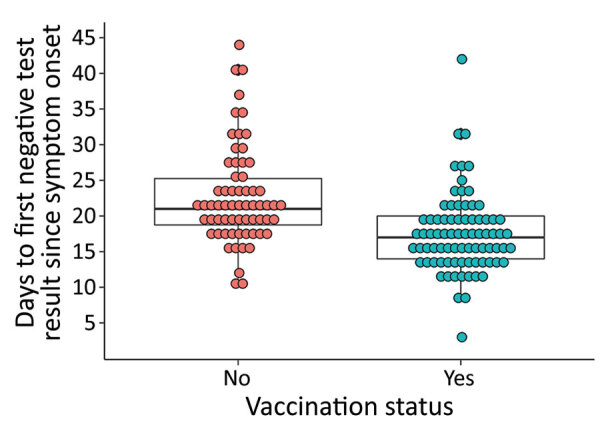
Days to first negative test result since symptom onset among patients who survived, stratified by vaccination status, n = 144, for impact of recombinant vesicular stomatitis virus–Zaire Ebola virus vaccination on Ebola virus disease illness and death, Democratic Republic of the Congo. Horizontal lines within boxes indicate medians; error bars indicate interquartile ranges, p<0.0001, by Wilcoxon rank sum test.

Vaccinated patients had a lower viral load, as indicated by a higher Ct value, than did unvaccinated patients (24.6 vs. 20.4; p<0.001) (Table 1). Among those who survived (n = 144), vaccinated patients cleared the virus more rapidly than did unvaccinated patients; this relationship was statistically significant and persisted when the data were analyzed using the date of symptom onset, first positive test result date, or date of admission to the ETC as the starting point (Figure 2; Appendix Figures 1, 2). Unvaccinated survivors of EVD also spent more time at the ETC than did vaccinated survivors (22.0 days vs. 20.0 days; p = 0.004).

### Deaths

Overall, 24.8% of vaccinated patients died, compared with 63.3% of unvaccinated patients (p<0.001). Previous vaccination with rVSV-ZEBOV was associated with decreased likelihood of death compared with those unvaccinated (odds ratio 0.19, 95% CI 0.12–0.30; p<0.001). This relationship persisted after controlling for potential confounders (adjusted odds ratio 0.26, 95% CI 0.15–0.46; p<0.001).

**Figure 3:**
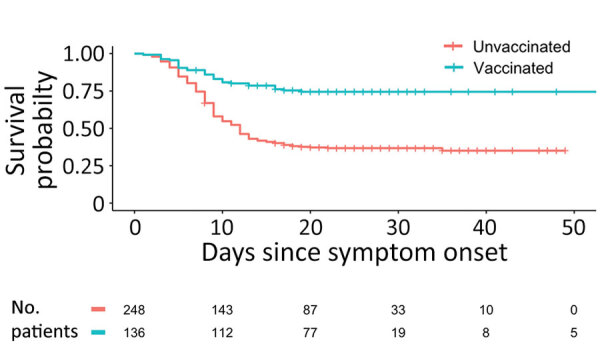
Kaplan-Meier survival plot of patients with Ebola virus disease, stratified by vaccination status, for study of effect of recombinant vesicular stomatitis virus–Zaire Ebola virus vaccination on Ebola virus disease illness and death, Democratic Republic of the Congo. Numbers below chart indicate number of ill patients at that time point, excluding patients who had died or who recovered and were discharged. One patient in the vaccinated group was excluded from this analysis because that patient did not have a reported date of discharge.

We used the Cox Proportional-Hazards model to determine the relationship between vaccination and death among all patients who had EVD symptom onset. After controlling for potential confounders, we found that vaccination remained a major predictor of reduced deaths for these patients (adjusted hazard ratio [aHR] 0.38, 95% CI 0.25–0.56) (Figure 3).

We also explored the relationship between timing of vaccination and death by using the Cox Proportional-Hazards Model for different subsets of all patients who were vaccinated. Models controlled for potential confounders. Vaccination with rVSV-ZEBOV reduced the risk for death in those vaccinated >10 days before symptom onset (aHR 0.41, 95% CI 0.23‒0.73; p = 0.002) and in those vaccinated <10 days before symptom onset (aHR 0.34, 95% CI 0.21–0.55; p<0.001) when compared with those unvaccinated. We developed a Kaplan-Meier curve for these data (Appendix Figure 3). These relationships persisted when using vaccination at >7 days and >14 days before symptom onset as cutoffs. Moreover, among those vaccinated >10 days before symptom onset, the specific number of days between vaccination and symptom onset was not a significant predictor of risk for death. This result was also true for those vaccinated <10 days before symptom onset.

We also explored the relationship between timing of vaccination and death by using the Cox Proportional-Hazards Model for different subsets of all patients who were vaccinated. Models controlled for potential confounders. Vaccination with rVSV-ZEBOV reduced the risk for death in those vaccinated >10 days before symptom onset (aHR 0.41, 95% CI 0.23‒0.73; p = 0.002) and in those vaccinated <10 days before symptom onset (aHR 0.34, 95% CI 0.21–0.55; p<0.001) when compared with those unvaccinated. We developed a Kaplan-Meier curve for these data (Appendix Figure 3). These relationships persisted when using vaccination at >7 days and >14 days before symptom onset as cutoffs. Moreover, among those vaccinated >10 days before symptom onset, the specific number of days between vaccination and symptom onset was not a significant predictor of risk for death. This result was also true for those vaccinated <10 days before symptom onset.

An additional 8 persons were vaccinated after symptom onset. Although these patients were not included in the larger analysis, we used the Cox Proportional-Hazards model to assess the effect of vaccine administration after symptom onset on death. The association between death and vaccination after symptom onset was not statistically significant (HR 0.22, 95% CI 0.03–1.61; p = 0.138).

## Discussion

In this study, we found that both preexposure and postexposure vaccination with rVSV-ZEBOV was associated with a reduction in EVD symptoms and deaths in laboratory-confirmed, EVD-positive patients. Vaccinated patients had a lower viral load upon admission and had fewer EVD-associated symptoms overall than their unvaccinated counterparts. Vaccinated persons were slightly older and more likely to have reported contact with a suspected or confirmed EVD-positive person. Unvaccinated persons were more likely to be female. Vaccinated persons also came to the ETC earlier in their disease course than unvaccinated patients, which might suggest that this population is more able or willing to engage with the healthcare system or to follow recommended health guidelines. Accepting the vaccine suggests more knowledge about the disease itself and is a positive health-seeking behavior; both of these factors might prompt such a person to seek care earlier. Increased knowledge of a disease has also been associated with increased vaccine uptake for other illnesses, including SARS-CoV-2 (*35*,*36*).

The willingness of vaccinated patients to seek care earlier in the disease course enabled treatment to be initiated earlier, which might have prevented their illness from becoming as severe as it otherwise might have been. Persons enrolled in the PALM Trial demonstrated similar behavior trends (*29*). Vaccinated persons were more likely to enroll in the trial sooner after the onset of symptoms, which, the authors concluded, might suggest a possible positive relationship between vaccination status and health-seeking behaviors. Data from the PALM Trial also highlight the need for initiation of treatment with mAb114 or REGN-EB3 early in the disease course. The authors observed an 11% increase in the odds of death for each additional day that symptoms persisted before enrollment in the study (*29*). In our study population, vaccinated persons were more likely to receive mAb114 or REGN-EB3 than unvaccinated persons. This finding might also positively impact illness and death. However, after controlling for treatment with experimental therapeutic agents in our model, we found that vaccination remained a major predictor of survival.

Our findings are consistent with results from a previous retrospective cohort study that also examined the impact vaccination had on EVD deaths in eastern DRC (*37*). Those authors concluded that EVD-positive persons who received rVSV-ZEBOV vaccine before admission had reduced viral load and reduced deaths compared with those who did not receive the vaccine. The authors also controlled for known EVD contact in their models. When we included this additional variable in our models, all relationships between the vaccine and deaths were preserved.

Vaccinated persons cleared the virus faster and had a shorter length of stay at the ETC than their unvaccinated counterparts, suggesting that they recovered faster from the disease. Because some patients were directed to the convalescent ward after 2 consecutive negative EVD test results, instead of being discharged to home, length of stay might be increased for the unvaccinated and vaccinated groups. However, we have no reason to believe that either group was preferentially sent to the convalescent ward.

After controlling for potential confounders, we found that vaccination with rVSV-ZEBOV before symptom onset was associated with decreased deaths. This relationship persisted regardless of timing of vaccine administration before onset of symptoms. These results suggest that the vaccine might still be effective days after exposure to EVD and that the extent of its effectiveness against death is not singularly dependent on timing of vaccination before symptom onset. The exact amount of time to complete vaccine-induced immune protection against EVD remains unclear (*38*); however, animal studies conducted in cynomolgus macaques demonstrated complete protection against EVD when the vaccine was administered 7 days before challenge and partial protection when administered 3 days before challenge (*39*). Thus, more aggressive vaccination campaigns in outbreak situations could be beneficial, especially given the observed reduced time to viral load clearance and shortened length of stay for hospitalized patients, in addition to the partial protection afforded by the vaccine in nonhuman primates.

Finally, only a small number of persons were vaccinated after symptom onset (n = 8). One died, and 7 survived. We were unable to conclude whether administration of the vaccine after symptom onset was also protective against EVD-associated illness and death. However, this finding is a potential avenue for future studies. Previous studies have explored the idea of using rVSV-ZEBOV as postexposure prophylaxis. In 1 study, rhesus macaques were infected with ZEBOV and subsequently vaccinated with rVSV-ZEBOV 24 hours postexposure. Results demonstrated that 33%–67% of the vaccinated animals survived infection (*40*). The vaccine has also been used as an experimental postexposure prophylaxis in humans after high-risk occupational exposures. In 1 instance, a person who sustained an accidental needle stick during an animal study at a Biosafety Level 4 facility received the vaccine 48 hours after the injury. No evidence of infection was detected during her hospitalization, and she was discharged from the hospital on day 21 (*41*). The vaccine has also been administered to clinical and nonclinical ETC staff after high-risk exposures; all staff had self-limited symptoms, including fever, after receiving the vaccine, and none showed development of EVD (*42**–**44*).

Further research into the potential role rVSV-ZEBOV might play in EVD treatment protocols is needed. More specifically, additional research is needed to evaluate the potential harmful interaction that could occur with coadministration of rVSV-ZEBOV vaccine, which is designed to elicit a neutralizing immune response to the main EBOV glycoprotein, and therapeutic mAbs, including REGN-EB3, which target the same glycoprotein (*45*). If administration of rVSV-ZEBOV vaccine alone or in combination with other therapeutics is shown to be effective on a larger scale as a treatment modality, this administration might have major implications with respect to the public health response and treatment for EVD outbreaks.

Much of the data used in this study were self-reported by patients, including their symptoms, recent contact with a suspected or confirmed EVD-positive person, and vaccination status. This self-reporting could lead to desirability bias with respect to vaccination status, as well as recall bias, particularly with respect to date of onset of symptoms and date of vaccination. In addition, there was missing data for a few variables, such as vaccination status, vaccination date, date of symptom onset, and final outcome. Removing patients who had missing data for these variables could lead to potential bias in the estimation of various parameters. Moreover, we used the Ct value as a proxy for viral load in the interpretation of our analysis. Although we attempted to adjust for confounders that might impact death and be associated with vaccination status, there are inevitably additional factors, including health literacy and health-seeking behaviors, which we were not able to adjust for in this study.

In conclusion, our results showed that previous vaccination with rVSV-ZEBOV reduces EVD-associated illness and death. This relationship persists regardless of vaccination timing, provided it is administered before onset of symptoms. This study directly addresses the paucity of scientific research identified by WHO as a limitation to rVSV-ZEBOV vaccine achieving full authorization for use in preventing EVD illness and death.

Appendix Additional information on effect of recombinant vesicular stomatitis virus–Zaire Ebola virus vaccination on Ebola virus disease illness and death, Democratic Republic of the Congo.
