# Supplementary material for: Effect of Recombinant Vesicular Stomatitis Virus–Zaire Ebola Virus Vaccination on Ebola Virus Disease Illness and Death, Democratic Republic of the Congo
Source: Emerg Infect Dis. 2022 Jun;28(6):1180–8. doi: 10.3201/eid2806.212223 (PMC9155898; doi:10.3201/eid2806.212223)
Supplement: Appendix — Additional information on effect of recombinant vesicular stomatitis virus–Zaire Ebola virus vaccination on Ebola virus disease illness and death, Democratic Republic of the Congo. [file 21-2223-Techapp-s1.pdf]

Article DOI: https://doi.org/10.3201/eid2806.212223

Effect of Recombinant Vesicular Stomatitis Virus–Zaire Ebola Virus Vaccination on Ebola Virus Disease Illness and Death, Democratic Republic of the Congo

Appendix

Study Procedures

Detailed information was collected about each patient on standardized clinical forms. Protocol based-care was provided to patients admitted to the Ebola Treatment Center (ETC). This care included monitoring and documentation of vital statistics, maintenance of electrolyte fluid levels, treatment for malaria, use of empiric antimicrobial drugs and intravenous fluids when needed, nutritional support, and basic mental health and psychosocial support (*1*). Ward rounds were performed by clinicians up to 6 times/day. The patient's clinical course was updated daily during their stay at the ETC, and their final disposition was also documented. Daily clinical laboratory testing was conducted, and patients were discharged from the ETC after 2 consecutive negative laboratory test results.

Data Management

Data were retrospectively abstracted from clinical documentation by independent trained study personnel blinded to the specific study aims and entered into a standardized digital database. As performed in previous Ebola virus disease (EVD) research, the process used digitalization of the medical charts in the Democratic Republic of the Congo, which were then deidentified in the abstraction process (*2,3*). Data sources for data abstraction included the patient log of all patients admitted to the Mangina ETC, which contained clinical and sociodemographic information for EVD-positive and EVD-negative persons. For EVD-positive patients, expanded abstraction was completed based on all available ETC medical documentation from the standardized case report forms during admission. To evaluate data abstraction accuracy, 15% (62) of the source records were double-entered by additional study personnel into a second database, and the databases were compared for discrepancies. Each discrepancy was recorded as

an error. The overall agreement across both databases was calculated and found to be in agreement for 97% of data points (Appendix Table 2).

In addition, the line list and more detailed EVD-positive database were compared across 145 variables for any discrepancies. For any flagged fields, paper charts were referenced for further clarification and resolution.

References

1. International Medical Corps. Viral hemorrhagic fever draft guidelines: standard clinical/psychosocial procedures for Ebola treatment unit (ETU) operations [cited 2022 Apr 1]. https://internationalmedicalcorps.org

2. Roshania R, Mallow M, Dunbar N, Mansary D, Shetty P, Lyon T, et al. Successful implementation of a multicountry clinical surveillance and data collection system for Ebola virus disease in West Africa: findings and lessons learned. Glob Health Sci Pract. 2016;4:394–409. PubMed https://doi.org/10.9745/GHSP-D-16-00186

3. Skrable K, Roshania R, Mallow M, Wolfman V, Siakor M, Levine AC. The natural history of acute Ebola Virus Disease among patients managed in five Ebola treatment units in West Africa: A retrospective cohort study. PLoS Negl Trop Dis. 2017;11:e0005700. PubMed https://doi.org/10.1371/journal.pntd.0005700

Appendix Table 1. Specific Ebola virus disease therapeutic received by vaccinated patients, stratified by vaccination timing*

Therapy	Vaccinated <7 d before symptom onset, n = 67	Vaccinated ≥7 d before symptom onset, n = 70
MAb114	20 (29.9)	26 (37.1)
REGN-EB3	30 (44.8)	19 (27.1)
Remdes	10 (14.9)	17 (24.3)
Zmapp	1 (1.5)	2 (2.9)
None	6 (8.9)	6 (8.6)

*Values are no.(%). MAb, monoclonal antibody.

Appendix Table 2. Quality of data entered from original patient charts into Ebola treatment Center database, n = 62

Case report form	Data entered correctly, %
Admissions	96.7
Triage Form 1	96.3
Triage Form 2	98.9
Triage Form 3	99.4
Laboratory Form	97.2
Overall	97.3

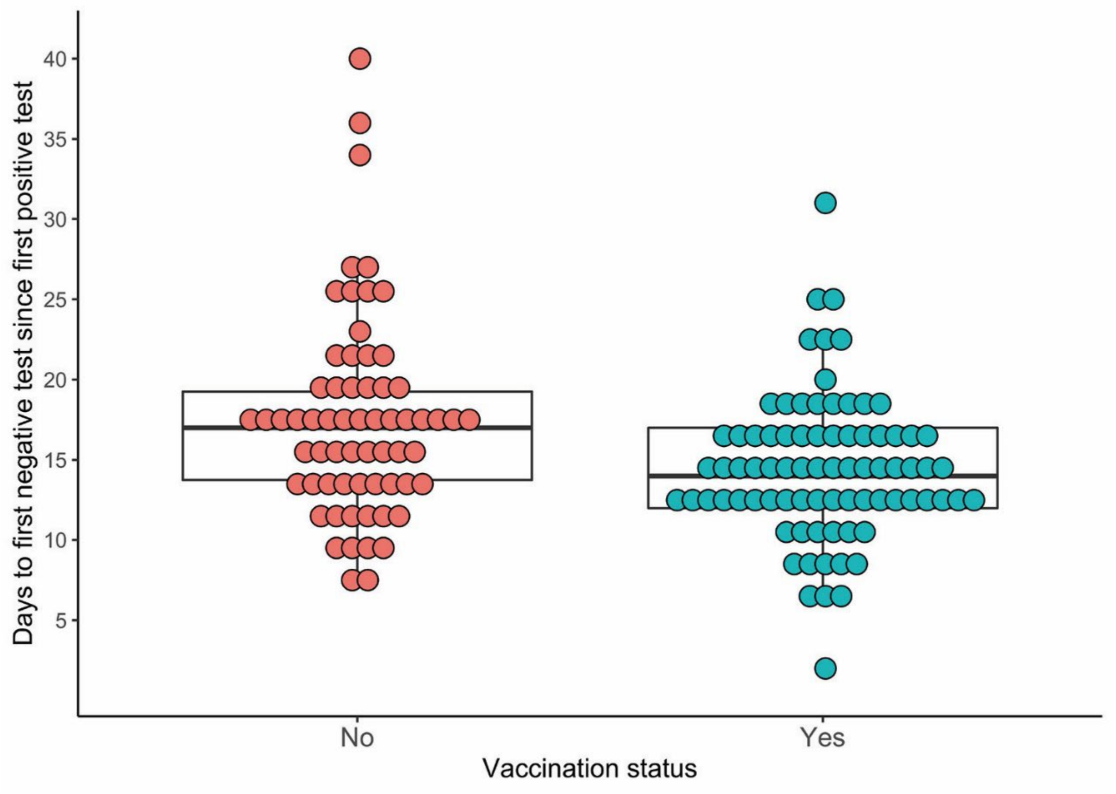

Appendix Figure 1. Days to first negative test since first positive test among patients who survived, stratified by vaccination status, N = 144. Box plot indicates median and interquartile range. p = 0.00098, by Wilcoxon rank sum test.

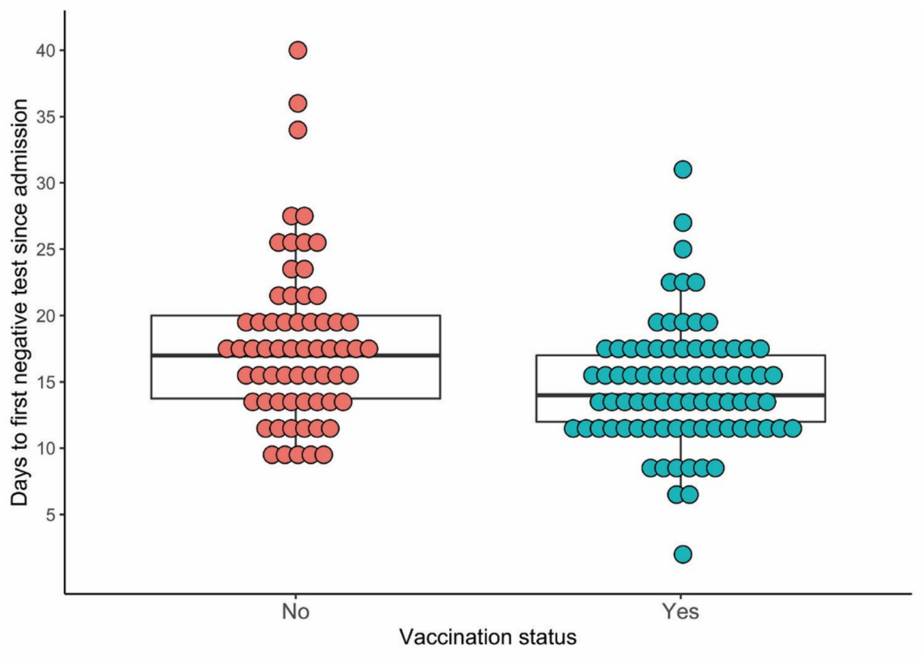

Appendix Figure 2. Days to first negative test since admission among patients who survived, stratified by vaccination status, N = 144. Box plot indicates median and interquartile range. p = 0.00048, by Wilcoxon rank sum test.

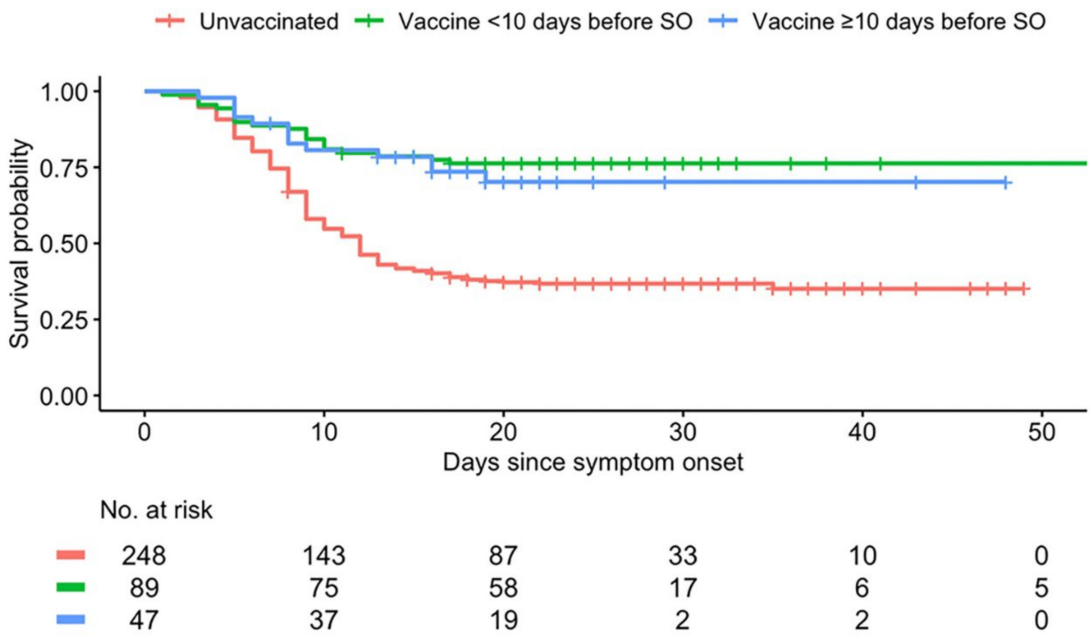

Appendix Figure 3. Kaplan-Meier survival plot of patients with Ebola Virus Disease, stratified by vaccination status and timing of vaccination before SO. One patient in the vaccinated <10 days before SO group was excluded from this analysis because they did not have a reported date of discharge. SO, symptom onset.
